# Rapid Response to Evaluate the Presence of Amphibian Chytrid Fungus (*Batrachochytrium dendrobatidis*) and Ranavirus in Wild Amphibian Populations in Madagascar

**DOI:** 10.1371/journal.pone.0125330

**Published:** 2015-06-17

**Authors:** Jonathan E. Kolby, Kristine M. Smith, Sara D. Ramirez, Falitiana Rabemananjara, Allan P. Pessier, Jesse L. Brunner, Caren S. Goldberg, Lee Berger, Lee F. Skerratt

**Affiliations:** 1 One Health Research Group, College of Public Health, Medical, and Veterinary Sciences, James Cook University, Townsville, Queensland, Australia; 2 EcoHealth Alliance, New York, New York, United States of America; 3 Sustainability Studies Program, Ramapo College of New Jersey, Mahwah, New Jersey, United States of America; 4 IUCN SSC Amphibian Specialist Group-Madagascar, Antananarivo, Madagascar; 5 Amphibian Disease Laboratory, Institute for Conservation Research, San Diego Zoo Global, San Diego, California, United States of America; 6 School of Biological Sciences, Washington State University, Pullman, Washington, United States of America; 7 School of the Environment, Washington State University, Pullman, Washington, United States of America; University of South Dakota, UNITED STATES

## Abstract

We performed a rapid response investigation to evaluate the presence and distribution of amphibian pathogens in Madagascar following our identification of amphibian chytrid fungus (*Batrachochytrium dendrobatidis*, *Bd*) and ranavirus in commercially exported amphibians. This targeted risk-based field surveillance program was conducted from February to April 2014 encompassing 12 regions and 47 survey sites. We simultaneously collected amphibian and environmental samples to increase survey sensitivity and performed sampling both in wilderness areas and commercial amphibian trade facilities. *Bd* was not detected in any of 508 amphibian skin swabs or 68 water filter samples, suggesting pathogen prevalence was below 0.8%, with 95% confidence during our visit. Ranavirus was detected in 5 of 97 amphibians, including one adult *Mantidactylus cowanii* and three unidentified larvae from Ranomafana National Park, and one adult *Mantidactylus mocquardi* from Ankaratra. Ranavirus was also detected in water samples collected from two commercial amphibian export facilities. We also provide the first report of an amphibian mass-mortality event observed in wild amphibians in Madagascar. Although neither *Bd* nor ranavirus appeared widespread in Madagascar during this investigation, additional health surveys are required to disentangle potential seasonal variations in pathogen abundance and detectability from actual changes in pathogen distribution and rates of spread. Accordingly, our results should be conservatively interpreted until a comparable survey effort during winter months has been performed. It is imperative that biosecurity practices be immediately adopted to limit the unintentional increased spread of disease through the movement of contaminated equipment or direct disposal of contaminated material from wildlife trade facilities. The presence of potentially introduced strains of ranaviruses suggests that Madagascar's reptile species might also be threatened by disease. Standardized population monitoring of key amphibian and reptile species should be established with urgency to enable early detection of potential impacts of disease emergence in this global biodiversity hotspot.

## Introduction

Global amphibian biodiversity is threatened by multiple factors including the emerging infectious diseases caused by the spread of amphibian chytrid fungus (*Batrachochytrium dendrobatidis*, *Bd*) and ranaviruses [1–4]. Madagascar possesses a wealth of endemic amphibian biodiversity, with 292 species described and over 500 believed to exist [5]. Many species are currently jeopardized by habitat destruction, exploitation for the pet trade, and climate change, and fortunately *Bd* and ranavirus have not previously been officially identified in the nation's wild amphibian populations nor have enigmatic amphibian declines been reported [6,7]. The apparent absence of *Bd* and ranavirus in Madagascar is remarkable, as these pathogens have already been detected in dozens of countries globally, including those nearby in eastern mainland Africa, and their continued spread appears certain [8–10]. Furthermore, Madagascar possesses high amphibian species richness and the climatic suitability expected to allow both pathogens to thrive [11].

Nearly a decade of field surveys for *Bd* in Madagascar have failed to produce confirmed positive results [6,12,13], suggesting either pathogen absence, severely limited *Bd* distribution, or the presence of a highly divergent *Bd* lineage that fails to react in current diagnostic protocols. Nearly a thousand amphibians comprised of dozens of species were sampled in these previous efforts, and presuming pathogen absence, a National Monitoring Plan (NMP) was recently designed and implemented to detect the arrival of *Bd* [14]. This proactive NMP called for long-term biannual surveys at eight nationally distributed sites, targeting three predicted "indicator” species at each location to serve as *Bd* sentinels. The three targeted species vary by site, but collectively includes four species of *Mantidactylus*, five species of *Heterixalus*, *Mantella cowanii* and *Ptychadena mascareniensis*. These species were selected for their widespread distribution and/or abundance and their susceptibility to *Bd* infection; at least one species per site (*M*. *betsilianus*, *H*. *betsileo*, *P*. *mascareniensis*) was shown to be susceptible to *Bd* in laboratory exposure trials [14]. Monitoring efforts have thus far spanned several field seasons and Weldon et al. (2013) suggested cooperation between national and international efforts would further assist early detection.

Meanwhile, the threat of ranaviruses to the biodiversity of Madagascar has attracted little attention despite their potential to drive long-term amphibian declines [3,4] and for some strains to cause disease in both amphibians and reptiles [15,16]. While some ranaviruses might be endemic to Madagascar and express relatively minimal virulence to native wildlife, targeted field surveillance efforts to evaluate ranavirus presence in the country is lacking. Due to the potentially severe disease-associated declines following introduction of non-native pathogens, we herein employ a precautionary approach and assume historical absence of both *Bd* and ranavirus from Madagascar in the absence data that may suggest otherwise.

To assist pathogen detection efforts in Madagascar, we recently sampled a commercial shipment of live amphibians exported to the USA for both *Bd* and ranavirus presence. *Bd* was detected on 3 (0.5%) of 565 wild-collected frogs upon removal from their shipping container [17], for the first time suggesting *Bd* presence in Madagascar following an unconfirmed report in 2010 [18]. We also detected ranavirus in these exported amphibians (18 of 29 sampled), likewise demonstrating its presence in the country. Unfortunately, it was uncertain whether these pathogens originated in wild amphibian populations in Madagascar because non-Malagasy contamination within the export facility could have occurred if foreign material had previously been imported. Despite the ambiguous origin, these data provided the first confirmation of *Bd* and ranavirus presence in Madagascar and raised our concern that recent pathogen introductions may have occurred. We quickly orchestrated and performed a highly targeted surveillance project that applied multiple techniques with greater collective sensitivity than the current NMP in order to produce a snapshot of national *Bd* and ranavirus distribution.

## Materials and Methods

### Ethics

All amphibian handling and sample collection methods employed in this investigation were approved by the Malagasy Direction Generale Des Forets and Madagascar National Parks as part of an emergency rapid response program. This work was performed under research permit #048/14/MEF/SG/DGF/DCB.SAP/SCB provided by the Malagasy Direction de la Biodiversite et du Systeme des Aires Protegees and export permit #'s 080N-EA04/MG14 and 151c_EA04/MG14 issued by the Malagasy Ministere de l'Environment et des Forets.

### Study sites and survey design

Field surveys were performed in Madagascar from 12 February to 4 April 2014. We applied a risk-based approach to the selection of survey localities and determination of which amphibian species and individuals to sample [19,20]. Regions targeted for sampling included species with predicted likelihood of pathogen exposure or susceptibility, locations in proximity to wildlife trade centers where elevated transmission rates and pathogen spillover may occur, and areas with predicted optimal environmental conditions and climatic suitability for *Bd* survival (e.g. cool wet habitats at high altitudes) [11]. Amphibians species prioritized for sampling were those described to have prolonged exposure to permanent aquatic habitats [21]. A greater diversity of water-associated species were targeted than those included by the current NMP, as susceptibility to pathogens differs between species [22,23]. Likewise, susceptibility to infection and disease varies with ontogeny, and is often higher earlier in life [24–26], so we also included pre-metamorphic animals not previously sampled by NMP activities.

Our survey efforts were performed during Madagascar's warm rainy season when most amphibian species are active, breeding, and can be readily encountered in high abundance near water bodies. Although elevated temperatures threaten the survival of both *Bd* [27] and ranavirus [28], we specifically targeted cooler habitats where conditions favored pathogen survival. Further, we believed that the frequent physical contact and water exposure during this breeding season might increase rates of transmission, the amount of time pathogens can be shed into the water, the number of amphibians that could be easily located to create a large sample pool, and hence increase detectability. Environmental conditions were measured at each survey site at the time of sampling, including water and air temperature, pH, and relative humidity.

Adult, juvenile, and larval amphibians were sampled. Tadpoles were collected by dip net and sampled by day whereas adult and juvenile frogs were primarily captured by hand, both day and night. Sampling was performed in locations where it appeared we could collect adequate numbers of amphibians (>30) in a single session to increase detection likelihood and statistical power. Throughout this investigation, we visually examined all sampled amphibians for skin lesions or other abnormalities and remained vigilant for amphibian mortality events.

### Amphibian Swabbing

Nearly all amphibians included in this investigation were sampled for both *Bd* and ranavirus, although some were sampled only for *Bd*. Upon capture, each amphibian was first sampled for *Bd* with a sterile fine-tipped rayon swab (Medical Wire & Equipment Co. #MW113) following Hyatt et al. (2007) [29]. For adult and juvenile amphibians, the swab bud was drawn across the ventral surface of the animals' hands, feet and pelvic patch five times each. For larval amphibians, swab buds were twirled in the buccal cavity as per Retallick et al. (2006) [30]. All swab buds were snapped off into 2 mL vials containing 1 mL 70% ethanol. A fresh pair of Nitrile gloves was worn for each new amphibian handled and changed between every sample to prevent cross contamination.

Amphibians were then sampled for ranavirus following non-lethal methods [15,31]. Cloacal swabs were collected from adults by inserting sterile rayon-tipped swabs (Puritan Medical Products #P25-800R) into the cloaca and gently twirling the handle several times. For small adults, juveniles, and tadpoles, buccal swabs were collected by inserting the swab into the oral cavity and twirling. Swab buds were cut off into 2 mL vials containing 1mL 70% ethanol. Scissors were decontaminated by flaming for 5 seconds between samples. All animals were released upon completion of sampling.

It was not possible to identify tadpoles to species with certainty. Instead, oral structures and body morphology were examined and used to categorize the approximate number of species sampled per location. Variable environmental conditions (e.g. water temperature, available nutrition, disease presence), and/or injury, may be associated with deformities in a tadpole's keratinized oral structures [32,33], potentially introducing some uncertainty into our species categorizations. As prevalence of *Bd* differs between tadpole species [19,34], efforts were made to sample a diversity of species in their larval stage to improve the chance of detection.

### Tadpole water

Prior to being swabbed, tadpoles at 16 sites were held aside in a 650 mL bowl of local river water for an hour to allow for the release of disease particles. This water was then collected for filtration and all tadpoles were released, except for the subsample that was then swabbed for *Bd* and ranavirus. The number of tadpoles present in each water sample and approximate number of species was recorded upon release. We performed this method to increase *Bd* and ranavirus detection probability by collecting and concentrating the pathogens off a greater number of animals than could be individually sampled.

A similar process was performed on one occasion with live crayfish (*Procambarus* spp.) in lieu of tadpoles, since *Bd* has been found within crayfish gastrointestinal tracts and these animals might serve as *Bd* reservoir hosts [35]. Approximately 500 crayfish collected by local fishermen at a site in Antananarivo were held in a large bucket, to which we added water from their habitat, and then allowed them to soak for 15 minutes. A sample of this water was then collected and filtered.

### Water Filtration

Water was sampled to detect the environmental presence of *Bd* and ranavirus following methods described by Goldberg et al. (2011) [36]. This method of sampling can capture microbial material either present independently in aquatic suspension or embedded in affected amphibian tissue cells shed into the water (environmental DNA, or "eDNA"). At aquatic survey sites, three 500 mL bottles of water were each collected approximately 20 m apart and then combined into one 1500 mL bottle before processing. Samples were collected near the top of the water column, between 5 and 10 cm below the surface. At commercial trade facilities where only one water bowl was present per enclosure housing a single species, single water collections were sampled, but where multiple species were housed communally, water was collected and combined from multiple enclosures to increase sampling efficiency. Water temperature and pH were measured with a portable handheld meter (Hannah Instruments #HI98128) at the middle of each sample locality.

Water was passed through 0.45μm Metricel (mixed cellulose esters) membrane filters held within sterile disposable filter funnels (Pall Corporation #4815) using a vacuum hand pump connected to a 1 L vacuum flask. In some instances, a battery-operated peristaltic pump was used in place of the vacuum hand pump when working with higher sample volumes, although the two methods seemed to be similarly effective and efficient. Water was processed until the filter paper became nearly clogged with debris and the flow significantly diminished, or until the total volume collected was filtered. The volume of water filtered was recorded and the filter membrane was removed with sterile forceps, folded inwards three times, and placed into a 50 mL sample tube. Each tube had a small hole in the cap covered with a pad of sterile gauze to allow air exchange, and was stored in a zip-top bag of silica gel beads to remove remaining moisture from the sample. Forceps were soaked in full-strength commercial bleach (6% sodium hypochlorite) to denature any contaminant DNA [37] and rinsed with filtered bottled spring water between samples. Water bottles used to collect the samples were also sterilized with this bleach solution and flushed with native water at each site three times immediately prior to sampling. This wastewater was discarded on land away from the water body. A fresh pair of Nitrile gloves was worn to handle each water filter sample. We sometimes processed multiple samples at a site to ensure adequate total volumes were filtered if rapid clogging of the filter membranes occurred. Water and amphibians were both concurrently sampled from the same habitat when possible, but water samples were always collected first before the team entered the water in order to prevent potential contamination and siltation of the sample.

### Field biosecurity

Biosecurity measures were strictly enforced throughout this investigation to prevent accidental spread of amphibian pathogens both between amphibians and locations [38]. Fresh pairs of Nitrile gloves and plastic bags used to hold frogs when sampling were each used only once and discarded. A bleach solution (10% commercial bleach) was used to rinse all materials exposed to amphibians or environmental substrates prior to leaving each study site (e.g. dip nets, water filtration equipment, footwear). Field boots were thoroughly scrubbed to remove all sediment prior to disinfection with this bleach solution.

### Sample Analysis

#### *Bd* swabs

Taqman PCR for *Bd* was based on the method, primers and probe of Boyle et al. 2004 [39]. The DNA template was prepared with Prepman Ultra (Applied Biosystems) and extractions were diluted 1:10. Reactions used the Taqman Environmental Mastermix 2.0 (Applied Biosystems). Samples were run in triplicate on an Applied Biosystems 7900HT thermocycler using 384 well plates with an exogenous internal positive control labeled with VIC (Applied Biosystems) for each sample to detect PCR inhibitors. Samples that amplified at a Ct≥50 and those without amplification in any of the wells were scored as negative. Quantification standards were created by growing *Bd* isolate JEL 197 on 1% tryptone agar and harvested of zoospores by rinsing plates with 1X PBS. After collection, zoospores were counted three times on a hemocytometer to determine a range of zoospores ml^-1^. Standard curves were generated with ten-fold serial dilutions (range 1 x 10^6^ to 1 x 10^–2^ zoospores). In addition to positive controls (quantification standards), each plate included a negative control (Taqman mastermix and no sample DNA) as well as 4 positive and negative quality assurance controls consisting of swabs either inoculated with *Bd* zoospores or sham-inoculated.

#### Ranavirus swabs

Swabs were first inverted in tube so that the bud was above the ethanol and then centrifuged at 13000 rpm for 5 minutes to pellet all of the suspended material. The ethanol was carefully removed with a pipette and then 100 μL of Prepman Ultra (Applied Biosystems) was added to the swab. Samples were then centrifuged again for 2 min, the swab removed, and then incubated at 100°C for 15 min according to manufacturer’s instructions. The sample was centrifuged again for 3 min and 20 μL of the supernatant containing the DNA was moved to a new sterile 1.5 mL snap cap tube and frozen until it could be screened.

The concentration of extracted DNA was measured using a NanoDrop-2000 (Thermo-Scientific) and, if necessary, diluted to approximately 20 ng DNA/μL. Extracted DNA from each sample was run full-strength and screened for ranavirus in triplicate 20 μL reactions on 96-well plates with 5 μL of DNA template (~100 ng) using a Taqman realtime polymerase chain reaction (qPCR) with primers and probe that amplify a 70-bp region within the major capsid protein of all known ranaviruses [40]. A 10-fold serial dilution of DNA extracted from a Frog Virus 3-like ranavirus grown in *Epithilium papilloma cyprinia* cells from 10^2^ to 10^7^ plaque-forming units (pfu's) was used as a standard against which unknown samples were quantified. Samples with amplification in two or three wells were scored as positive. Those without amplification in any of the wells were scored as negative. Ambiguous samples were re-run and if at least one well showed amplification in the second run, the sample was scored as positive. We used an Exogenous Internal Positive Control (Exo IPC, Applied Biosystems) in the third well of each sample to detect PCR inhibition of DNA, potentially caused by matter from the environment or the PrepMan Ultra (Applied Biosystems). If inhibition was detected the sample was diluted 1:10 and re-run. Viral quantities for positive samples are reported as the mean of the log_10_ (pfu) across all wells of the sample (i.e. including any zeros).

#### Water filters for *Bd* and ranavirus

We extracted DNA from filters using the QIAshredder/DNeasy Blood and Tissue DNA extraction kit method described in Goldberg et al. (2011) [36], in a room where no high-quality DNA extracts or PCR products had been handled and where researchers were required to shower and change clothing before entering if they had previously been in a room with PCR product. An extraction negative was created with each set of extractions and negative and positive PCR controls were included in each plate. All samples were run in triplicate. We tested for *Bd* using the assay of Boyle et al. (2004) [39] with a 6FAM-labeled probe and for ranavirus using the assay of Picco et al. (2007) [40], as above but with a NED-labeled probe, in a multiplex reaction. As part of multiplex validation, three known positive tissue samples for each pathogen were quantified in this reaction singly and in combination; Cq values were within 0.5 for each sample. Reactions were run using Quantitect Multiplex PCR Mix (Qiagen, Inc.) with recommended multiplexing concentrations (1X QuantiTect Multiplex PCR mix, 0.2 μM of each primer, and 0.2 μM of each probe) on an Applied Biosystems 7500 Fast Real-Time PCR System. Reactions were 15 μL in volume and each included 3 μL of sample. Cycling began with 15 min at 95°C followed by 50 cycles of 94°C for 60 s and 62°C for 60 s and went for 50 cycles. All reactions included an internal positive control (IC; Qiagen, Inc.). Samples showing inhibition (>3 Ct difference compared with the negative controls) were processed through a OneStep PCR Inhibitor Removal Kit (Zymo Research Corp.) and rerun in triplicate. Samples that tested positive in <3 wells in the first run were rerun in triplicate; if at least one well tested positive in each plate on the second run, that sample was considered positive.

## Results

### Field Surveys

We sampled a total of 508 amphibians for pathogen detection via swabbing, including 483 free-ranging amphibians and 25 wild-collected amphibians sampled at a commercial wildlife export facility in Antananarivo (Table 1). Metamorphs and adults of 37 species were sampled. Field identification of some adult animals was only possible to genus and we conservatively grouped these animals together into a single genus-level record in our results. The number of species of larvae we sampled is not known due to challenges with identification. Therefore, a greater diversity of amphibian species may have been included in this survey than expressed. All amphibian species sampled are endemic to Madagascar with the exception of the country's two exotic introduced species, *Hoplobatrachus tigerinus* and *Duttaphrynus melanostictus*.

**Table 1:** Site summary and diversity of amphibians sampled for the presence of *Batrachochytrium dendrobatidis* (*Bd*) and ranavirus in Madagascar.

Region	Site	Desc.	Lat(S)	Long(E)	Altitude(m)	A	W	Species	T
**Andasibe**	1	River	18.935	48.413	952	23.6	19.2	*Anodonthyla boulengeri Boophis luteus Boophis madagascariensis Heterixalus betileo Mantidactylus betsileanus Mantidactylus femoralis Mantidactylus grandidieri Mantidactylus melanopleura Spinomantis aglavei*	0
**Andasibe**	2	Pond	18.933	48.413	936	N/A	N/A	*Mantidactylus betsileanus Ptychadena mascareniensis*	0
**Andringitra**	1	River	22.144	46.888	1729	23.1	18.5	*Mantidactylus spp*.	1
**Andringitra**	2	River	22.162	46.895	2050	21.4	20.5	*Mantidactylus spp*. *Ptychadena mascareniensis*	2
**Andringitra**	3	River	22.130	46.866	2111	20.5	23.9	*Boophis microtympanum Mantidactylus spp*.	1
**Andringitra**	4	River	22.153	46.900	1968	N/A	N/A	*Boophis goudoti Boophis microtympanum*	0
**Ankarafantsika**	1	Pond	N/A	N/A	N/A	31.4	28.4	*Laliostoma labrosum Ptychadena mascareniensis*	1
**Ankarafantsika**	2	River	16.326	46.857	125	30.7	25.9	*Mantidactylus spp*. *Ptychadena mascareniensis Stumpffia spp*.	0
**Ankarafantsika**	3	Rice Paddy	16.343	46.848	92	31.8	31.5	*Boophis spp*. *Ptychadena mascareniensis*	1
**Ankaratra**	1	River	19.333	47.263	2384	12.4	13.4	*Boophis williamsi Mantidactylus curtus Mantidactylus spp*.	1
**Ankaratra**	2	River	19.349	47.279	2032	13.3	13.9	*Mantidactylus curtus Mantidactylus mocquardi Mantidactylus spp*.	1
**Ankaratra**	3	River	19.346	47.279	2015	15.9	14.3	*Mantidactylus mocquardi Mantidactylus pauliani*	3
**Antananarivo**	1	Rice Paddy	18.861	47.435	1249	30.2	25.6	*Ptychadena mascareniensis*	0
**Antananarivo** ^*^	2	Trade Facility	18.785	47.463	1286	N/A	N/A	*Dyscophus guineti Heterixalus madagascariensis Scaphiophryne madagascariensis*	0
**Isalo**	1	River	N/A	N/A	N/A	28.3	24.1	*Mantidactylus spp*. *Ptychadena mascareniensis*	0
**Isalo**	2	River	22.628	45.359	801	32.8	25.2	*Mantidactylus spp*. *Ptychadena mascareniensis*	0
**Isalo**	3	River	22.645	45.332	792	34.2	26.5	*Blommersia spp*. *Mantidactylus spp*. *Ptychadena mascareniensis*	1
**Ranomafana**	1	River	N/A	N/A	N/A	21.4	19.8	Unknown	2
**Ranomafana**	2	River	21.254	47.421	932	24.4	19.1	*Mantidactylus betsileanus Mantidactylus mocquardi*	2
**Ranomafana**	3	River	21.269	47.425	992	22.1	20.0	*Mantidactylus betsileanus Mantidactylus cowanii Mantidactylus majori Mantidactylus melanopleura*	3
**Ranomafana**	4	River	21.291	47.426	1052	23.1	19.5	*Mantidactylus cowanii Mantidactylus femoralis Mantidactylus majori*	2
**Ranomafana**	5	River	21.291	47.426	1053	22.3	19.6	*Mantidactylus cowanii Mantidactylus grandidieri Mantidactylus majori Mantidactylus spp*.	3
**Toamasina**	1	Pond	18.149	49.375	10	24.3	26.2	*Bufo melanostictus Hoplobatrachus tigerinus*	0
**Zahamena**	1	River	17.513	48.726	1072	20.1	19.2	*Mantidactylus sp*.	0
**Zahamena**	2	River	17.500	48.734	1202	18.8	17.7	*Boophis boehmi Gephyromantis moseri Mantidactylus albofrenatus Mantidactylus charlotteae Plethodontohyla notostica*	1
**Zahamena**	3	River	17.508	48.731	1068	21.0	18.2	*Boophis liami Boophis picturatus Mantella nigricans Mantidactylus charlotteae Mantidactylus femoralis Mantidactylus grandidieri Mantidactylus lugubris Mantidactylus mocquardi*	1

Air (A) and water (W) temperatures measured at the sample site in degrees Celsius. Approximate number of species of larval amphibians (T) included in the sample at that location.

*Amphibians sampled in Antananarivo but reported to have been collected from the wild in Fierenana.

**Fig 1:**
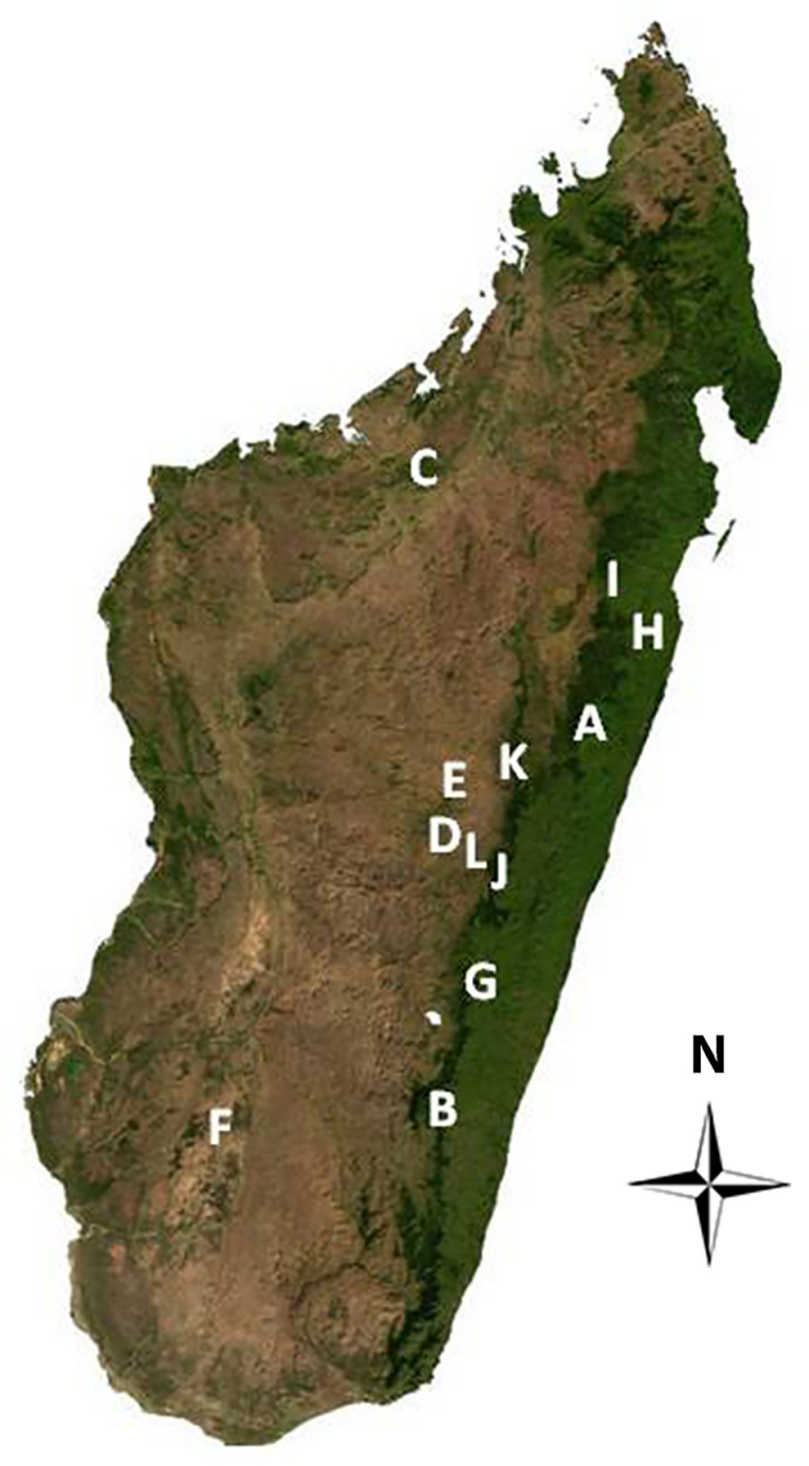
Locations sampled for the presence of *Bd* and ranavirus in Madagascar. Ambatolampy (A), Analamay (B), Andasibe (C), Andringitra National Park (D), Ankarafantsika (E), Ankaratra (F), Antananarivo (G), Faravohitra (H), Isalo (I), Ranomafana National Park (J), Toamasina (K), Zahamena National Park (L). The base map was obtained from www.maplibrary.org. GPS coordinates were used to identify locations on Google Earth (Google Inc., 2013) and edited onto the base map with Adobe PhotoShop CS6 (Adobe, 2012).

Twelve regions were surveyed, spanning approximately 750 km from Ankarafantsika in the North to Isalo in the South, and eastward along the country's rainforest belt (Fig 1). Between one and five separate water bodies were sampled in each region of intensive sampling, with three sampled in most instances. In total, we sampled 45 water bodies via water filtration. Volumes processed per filter ranged from 75 mL to 6000 mL, but most samples were highly turbid and clogged the filters at lower volumes (≤500 mL). In total, 66.30 L of environmental water and 4.25 L of tadpole water were filtered.

### *Bd* Swabs

All 508 amphibians swabbed for *Bd* detection tested negative for the presence of *Bd* by qPCR and showed no obvious signs of disease (Table 2), apart from two tadpoles with severe oral deformities.

**Table 2:** Detection of *Batrachochytrium dendrobatidis* (*Bd*) in Madagascar by quantitative PCR (qPCR) and apparent prevalence including 95% confidence limits (CL) at each location as per swab results, assuming *Bd* qPCR sensitivity and specificity as per Skerratt et al. (2011) of. 729 and. 942, respectively.

Region	Site	Prevalence (95% CI)	Water	No. Sampled	No. *Bd*+	#Spp.	Tadpole/ Adult	#Tad
Andasibe	1	0 (0–0.184)	-	17	0	9	A	0
Andasibe	2	0 (0–0.194)	-	16	0	2	A	0
**Andasibe Total**		**0 (0**–**0.104)**	-	**33**	**0**	**10**	**A**	**0**
Andringitra	1	0 (0–0.138)	-	24	0	1	T+A	15
Andringitra	2	0 (0–0.114)	-	30	0	2	T+A	15
Andringitra	3	0 (0–0.133)	-	25	0	2	T+A	17
Andringitra	4	0 (0–0.259)	-	11	0	2	A	0
**Andringitra Total**		**0 (0**–**0.041)**	**-**	**90**	**0**	**4**	**T+A**	**47**
Ankarafantsika	1	0 (0–0.155)	-	21	0	2	T+A	15
Ankarafantsika	2	0 (0–0.278)	-	10	0	3	A	0
Ankarafantsika	3	0 (0–0.138)	-	24	0	2	T+A	14
**Ankarafantsika Total**		**0 (0**–**0.065)**	**-**	**55**	**0**	**5**	**T+A**	**29**
Ankaratra	1	0 (0–0.242)	-	12	0	3	T+A	6
Ankaratra	2	0 (0–0.133)	-	25	0	3	T+A	6
Ankaratra	3	0 (0–0.114)	-	30	0	2	T+A	15
**Ankaratra Total**		**0 (0**–**0.054)**	**-**	**67**	**0**	**5**	**T+A**	**27**
Antananarivo*	1	0 (0–0.278)	-	25	0	1	A	0
Antananarivo	2	0 (0–0.133)	-	10	0	3	A	0
**Antananarivo Total**		**0 (0**–**0.099)**	-	**35**	**0**	**4**	**A**	**0**
Isalo	1	0 (0–0.354)	-	7	0	2	A	0
Isalo	2	0 (0 –0.299)	-	9	0	2	A	0
Isalo	3	0 (0–0.114)	-	30	0	3	T+A	8
**Isalo Total**		**0 (0**–**0.077)**	-	**46**	**0**	**3**	**T+A**	**8**
Ranomafana	1	0 (0–0.278)	-	10	0	1	T	10
Ranomafana	2	0 (0–0.143)	-	23	0	2	T+A	20
Ranomafana	3	0 (0–0.107)	-	32	0	4	T+A	20
Ranomafana	4	0 (0–0.161)	-	20	0	3	T+A	10
Ranomafana	5	0 (0–0.138)	-	24	0	4	T+A	12
**Ranomafana Total**		**0 (0**–**0.034)**	-	**109**	**0**	**8**	**T+A**	**72**
Toamasina	1	0 (0 –0.299)	-	9	0	2	A	0
**Toamasina Total**		**0 (0**–**0.299)**	-	**9**	**0**	**2**	**A**	**0**
Zahamena	1	0 (0–0.793)	-	1	0	1	A	0
Zahamena	2	0 (0–0.104)	-	33	0	5	T+A	21
Zahamena	3	0 (0–0.114)	-	30	0	8	T+A	13
**Zahamena Total**		**0 (0**–**0.057)**	-	**64**	**0**	**13**	**T+A**	**34**
**National Total**		**0 (0**–**0.008)**	**-**	**508**	**0**	**37**	**T+A**	**217**

Number of animals sampled, number of species represented, whether tadpoles (T) or post-metamorphic (A) animals were included, and number of tadpoles (#Tad) in the sample are reflected. Water filter results for *Bd* detection also presented. For sites where all amphibian swabs and water samples together tested negative for *Bd*, true prevalence is likely closer to the lower CL. Cumulative data for all sites sampled within the region appear in bold.

*Amphibians sampled in Antananarivo but reported to have been collected from the wild in Fierenana.

### Ranavirus Swabs

Of the 508 amphibians sampled for *Bd*, 499 were also swabbed for ranavirus. Due to current funding limitations a subset of 97 ranavirus samples were prioritized for analysis. Five of these 97 samples tested positive for the presence of ranavirus (prevalence = 5.2%; 95% CI: 2.2–11.5%). Four positive amphibians were sampled from three separate water bodies in Ranomafana National Park; one adult *Mantidactylus cowanii* (cloacal swab) and three unidentified larvae (buccal swabs). The other positive sample was from an adult *Mantidactylus mocquardi* from Ankaratra (Table 3). The titers from these positive swabs were quite low (range: 2.9–12.6 pfu equivalents) and none of the animals showed clinical symptoms of infection when sampled.

**Table 3:** Detection of ranavirus (RV) in Madagascar by quantitative PCR (qPCR) and apparent prevalence including 95% confidence limits (CL) for infection as per swab results, assuming qPCR sensitivity of. 80 as per Gray et al. (2012) and absolute specificity (1.0).

Location	Site	Prev (95% CI)	Water	No. Sampled	No. Rv+	#Spp.	Tadpole/Adult	#Tad
Andringitra	2	0 (0–0.354)	-	7	0	2	A	0
Andringitra	4	0 (0–0.434)	-	5	0	2	A	0
**Andringitra Total**		**0 (0**–**0.242)**	**-**	**12**	**0**	**4**	**A**	**0**
Ankaratra	1	0 (0–0.658)	-	2	0	1	T	2
Ankaratra	4	0.042 (0.002–0.203)	-	30	1	2	T+A	15
**Ankaratra Total**		**0.039 (0.002**–**0.202)**	**-**	**32**	**1**	**3**	**T+A**	**17**
Antananarivo*	2	0 (0–0.490)	**+**	4	0	2	A	0
**Antananarivo Total**		**0 (0**–**0.490)**	-	**4**	**0**	**2**	**A**	**0**
Ranomafana	2	0 (0–0.793)	-	1	0	1	T	1
Ranomafana	3	1 (0.062–1)	-	1	1	1	T	1
Ranomafana	4	0.100 (0.028–0.301)	-	20	2	3	T+A	10
Ranomafana	5	0.060 (0.003–0.284)	-	21	1	3	T+A	12
**Ranomafana Total**		**0.116 (0.040**–**0.269)**	-	**43**	**4**	**4**	**T+A**	**24**
Toamasina	1	0 (0–0.390)	-	6	0	2	A	0
**Toamasina Total**		**0 (0**–**0.390)**	-	**6**	**0**	**2**	**A**	**0**
**National Total**		**0.064 (0.026**–**0.144)**	**+**	**97**	**5**	**15**	**T+A**	**41**

Number of animals sampled, number of species represented, whether tadpoles (T) or post-metamorphic (A) animals were included, and number of tadpoles (#Tad) in the sample are reflected. Water filter results for RV detection also presented. For sites where all amphibian swabs and water samples together tested negative for RV, true prevalence is likely closer to the lower CL. Cumulative data for all sites sampled within the region appear in bold.

*Amphibians sampled in Antananarivo but reported to have been collected from the wild in Fierenana.

### Water Filters

Ranavirus was detected in the water bowls at two locations where wild-collected amphibians were temporarily held in captivity pending commercial exportation: one trade facility in Toamasina and the other in Antananarivo (Table 4). In the Toamasina facility, five amphibian enclosures were sampled; each held multiple amphibian species of the genera *Boophis*, *Heterixalus* and *Mantella* collected from unknown localities. Samples from enclosures that shared a screened wall were combined (i.e. 1 & 2, 3 & 4), producing a total of three samples. Two of three samples tested positive for ranavirus (i.e. 3 & 4 and 5). The water introduced to these enclosures was pumped on-site from an underground well. Samples of this well water tested negative for both ranavirus and *Bd*, suggesting ranavirus was introduced by infected wild-collected frogs rather than contaminated groundwater. This facility was the source of frogs previously found to be infected with *Bd* [17] and ranavirus, but very few frogs were present in these enclosures at the time of this water collection and were not included in our sampling. In Antananarivo, water from one of three enclosures, which held only tomato frogs (*Dyscophus guineti*), tested positive. Ranavirus was not detected in the other two enclosures sampled at this facility, one of which held *Heterixalus madagascariensis* and the other *Scaphiophryne madagascariensis*. The operator of this facility reported to JEK that all amphibians had been collected from Fierenana, near Moramanga. The source of water in these bowls was said to be rainwater collected onsite.

**Table 4:** Water filter samples processed by quantitative PCR for detection of *Batrachochytrium dendrobatidis* (*Bd*) and ranavirus (RV) in Madagascar.

Region	Site	Habitat/Source	Lat(S)	Long(E)	Air (C)	RH(%)	W(C)	pH	Swabs	W vol	T vol	T#	*Bd*	RV
**Ambatolampy**	1	Rice paddy	19.381	47.426	18.3	79.0	20.4	6.89	No	500 (1)	N/A	N/A	-	-
**Analamay**	1	Flooded dirt road	18.831	48.313	22.8	79.8	25.6	7.28	Yes	1800 (4)	N/A	N/A	-	-
**Andasibe**	1	River	18.935	48.413	23.6	82.8	19.2	7.46	Yes	600 (1)	N/A	N/A	-	-
**Andasibe (Mitsinjo amphibian breeding facility)**	3	River water provided to captive amphibians	18.933	48.413	21.4	81.8	21.4	7.79	No	1000 (1)	N/A	N/A	-	-
**Andasibe (Mitsinjo amphibian breeding facility)**	4	Water flushed from enclosures, (*Mantella aurantiaca*)	18.933	48.413	22.6	82.0	22.3	7.64	No	300 (1)	N/A	N/A	-	-
**Andasibe (Mitsinjo amphibian breeding facility)**	5	Water flushed from enclosures, *Boophis spp*. & *Mantidactylus spp*.)	18.933	48.413	22.9	82.0	21.8	7.5	No	500 (1)	N/A	N/A	-	-
**Andringitra**	1	River	22.144	46.888	23.1	63.1	18.5	6.51	Yes	3000 (1)	150 (1)	15	-	-
**Andringitra**	2	River	22.162	46.895	21.4	63.3	20.5	6.93	Yes	1500(1)	75 (1)	160	-	-
**Andringitra**	3	River	22.130	46.866	20.5	52.7	23.9	7.35	Yes	450 (1)	105 (1)	58	-	-
**Ankarafantsika**	1	Pond	N/A	N/A	31.4	80.4	28.4	7.04	Yes	700 (2)	95 (1)	209	-	-
**Ankarafantsika**	2	River	16.326	46.857	30.7	70.8	25.9	6.55	Yes	375(1)	N/A	N/A	-	-
**Ankarafantsika**	3	Rice paddy	16.343	46.848	31.8	73.4	31.5	6.89	Yes	1400 (2)	175 (1)	22	-	-
**Ankarafantsika (Durrell Chelonian Captive Breeding Centre)**	4	Concrete pools, Madagascan big-headed turtles (*Erymnochelys madagascariensis*)	16.313	46.817	33.9	69.8	32.0	7.04	No	550 (1)	N/A	N/A	-	-
**Ankarafantsika (Durrell Chelonian Captive Breeding Centre)**	5	Water bowls, Ploughshare tortoises (*Astrochelys yniphora*)	16.313	46.817	33.3	71.5	30.0	6.85	No	1100 (2)	N/A	N/A	-	-
**Ankaratra**	1	River	19.333	47.263	12.4	99.9	13.4	7.45	Yes	12000 (2)	425 (2)	6	-	-
**Ankaratra**	2	River	19.346	47.279	13.3	99.9	13.9	7.25	Yes	5000 (2)	250 (1)	6	-	-
**Ankaratra**	3	River	19.336	47.281	15.9	99.9	14.3	7.16	Yes	1450 (2)^*^	250 (1)	20	-	-
**Ankaratra**	4	River	19.349	47.279	15.7	91.1	15.2	7.31	No	4800 (2) ^*^	N/A	N/A	-	-
**Antananarivo**	1	Rice Paddy	18.861	47.435	30.2	65.2	25.6	6.89	Yes	600 (2)	300 (2)^c^	N/A	-	-
**Antananarivo**	2	Trade Facility (water bowl, *Heterixalus madagascariensis*)	18.785	47.463	24.4	87.8	23.4	6.92	Yes	150 (1)	N/A	N/A	-	-
**Antananarivo**	3	Trade Facility (water bowl, *Dyscophus guineti*)	18.785	47.463	24.9	92.1	22.2	7.04	Yes	450 (2)	N/A	N/A	-	**+**
**Antananarivo**	4	Trade Facility (water bowl, *Scaphiophryne madagascariensis*)	18.785	47.463	24.5	84.8	21.3	7.12	Yes	75 (1)	N/A	N/A	-	-
**Antananarivo**	5	Lake (crocodile farm outside trade facility)	18.785	47.463	22.8	68.8	23.0	7.54	No	700 (2)	N/A	N/A	-	-
**Antananarivo**	6	Lake #1 (Tsimbazaza Zoo)	18.930	47.527	20.7	72.0	22.0	7.2	No	600 (2)	N/A	N/A	-	-
**Antananarivo**	7	Lake #2 (Tsimbazaza Zoo)	18.931	47.526	21.7	64.8	21.3	7.31	No	600 (2)	N/A	N/A	-	-
**Faravohitra**	1	Lake #1 (outdoor trout aquaculture facility)	19.359	47.316	19.1	83.1	17.5	6.89	No	1200 (1)	N/A	N/A	-	-
**Faravohitra**	2	Lake #2 (outdoor trout aquaculture facility)	19.359	47.316	19.3	79.1	17.9	7.16	No	1000 (1)	N/A	N/A	-	-
**Faravohitra**	3	River (water supplied to trout aquaculture facility	19.359	47.316	19.6	75.3	16.09	7.02	No	1500 (1)	N/A	N/A	-	-
**Isalo**	1	River	N/A	N/A	28.3	55.9	24.1	6.92	Yes	2800 (2)	N/A	N/A	-	-
**Isalo**	2	River	22.628	45.359	32.8	37.5	25.2	7.00	Yes	1100 (2)	N/A	N/A	-	-
**Isalo**	3	River	22.645	45.332	34.2	28.8	26.5	7.01	Yes	1700 (2)	200 (1)	8	-	-
**Ranomafana**	1	River	N/A	N/A	21.4	95.0	19.8	7.22	Yes	1500 (1)	500 (1)	17	-	-
**Ranomafana**	2	River	21.254	47.421	24.4	75.8	19.1	7.02	Yes	1500 (1)	650 (2)	45	-	-
**Ranomafana**	3	River	21.269	47.425	22.1	90.2	20.0	7.02	Yes	1350 (1)	400 (2)	105	-	-
**Ranomafana**	4	River	21.291	47.426	23.1	81.5	19.5	6.97	Yes	1500 (1)	500 (2)	53	-	-
**Ranomafana**	5	River	21.291	47.426	22.3	89.3	19.6	6.96	Yes	1500 (1)	350 (2)	57	-	-
**Toamasina**	1	Flooded grass lot	18.149	49.375	24.3	93.8	26.2	6.84	Yes	600 (2) ^*^	N/A	N/A	-	-
**Toamasina**	2	Trade Facility (water bowls, enclosure #1 & 2)	18.147	49.401	n/a	n/a	n/a	n/a	No	250 (2)	N/A	N/A	-	-
**Toamasina**	2	Trade Facility (water bowls, enclosure #3 & 4)	18.147	49.401	n/a	n/a	n/a	n/a	No	600 (2)	N/A	N/A	-	**+**
**Toamasina**	2	Trade Facility (water bowl, enclosure #5)	18.147	49.401	n/a	n/a	n/a	n/a	No	400 (2)	N/A	N/A	-	**+**
**Toamasina**	2	Trade Facility (flooded grass lot)	18.147	49.401	n/a	n/a	n/a	n/a	No	1200 (2)	N/A	N/A	-	-
**Toamasina**	2	Trade Facility (well water supplied to enclosures)	18.147	49.401	n/a	n/a	n/a	n/a	No	3000 (1)	N/A	N/A	-	-
**Zahamena**	1	River	17.508	48.731	20.1	96.1	19.2	7.6	Yes	400 (1)	N/A	N/A	-	-
**Zahamena**	2	River	17.513	48.726	18.8	95.8	17.7	7.61	Yes	1500 (1)	325 (1)	21	-	-
**Zahamena**	3	River	17.500	48.734	21.0	88.3	18.2	7.74	Yes	1500 (1)	N/A	N/A	-	-

Environmental conditions measured at sample sites include air temperature, relative humidity, water temperature and pH. Total volume of environmental water (W vol) and tadpole water (T vol) filtered at each site is followed by the number of individual filter samples in parenthesis and number of tadpoles held in the Tvol sample (T#).

*Strong PCR inhibition was detected in these samples.

^c^Water held crayfish rather than tadpoles.

All water samples collected from natural amphibian habitats tested negative for the presence of both *Bd* and ranavirus by qPCR, although five samples from three locations demonstrated too much PCR inhibition for reliable testing, even after inhibitor removal. Environmental conditions at sampled locations fell mostly within the range suitable for *Bd* and ranavirus survival and reproduction, with air temperatures ranging from 12.4 to 34.2 C, averaging 23.3 C, and water temperatures from 13.4 to 32.0 C, averaging 21.5 C. Relative humidity was often high (average 78.3%) and water pH fluctuated little, ranging from 6.51 to 7.79, averaging 7.12. Only 3 of 45 water bodies displayed aquatic temperatures ≥ 30.0 C when sampled: one exposed rice paddy and two artificial bodies of water at a chelonian breeding facility, all located in Ankarafantsika.

## Discussion

The presence of both *Bd* and ranavirus in Madagascar appeared to be highly localized and at low prevalence during Feb-March 2014. We detected ranavirus (5 of 97), but not *Bd* (0 of 508), in amphibians and water samples (3 of 68). The prevalence of *Bd* was below 0.8% with 95% confidence at the time of sampling (0–0.8%), using Blaker's method [41] and the *Bd* diagnostic specificity and sensitivity values reported by Skerratt et al. (2011) [42], suggesting either *Bd* absence from sample localities or a failure to detect the pathogen if prevalence and/or infection intensity in amphibians were exceptionally low. For ranavirus, the nonlethal sampling method we employed can underestimate true prevalence by 20% compared to liver samples [31], so assuming our swabs had a reduced sensitivity of 0.80, we can be 95% confident that the cumulative prevalence of infection among those processed was 6.4% (2.6–14.4%) as per Blaker's method. Also considering the very low ranavirus loads detected, it is possible that some animals which tested negative may have carried infections loads that escaped our detection, suggesting our findings might further underestimate true prevalence and distribution. Ranavirus-positive amphibians were detected at four separate rivers, three within Ranomafana National Park and one in Ankaratra.

It is unclear precisely how water filter results for *Bd* and ranavirus detection relate to concurrent prevalence of infection in amphibians, but there are two studies from North America that are relevant. Hall et al. (submitted) [43] used similar methods to ours (but with 0.22 μm filters compared to our 0.45 μm pore size) in a survey of wood frog (*Rana sylvatica*) tadpoles in vernal pools in Connecticut, USA and estimated the probability of detecting ranavirus DNA at 0.90 per 250 mL filtered water sample in ponds with known infection (note that all tadpoles tested from their ponds were infected). Assuming the same detection probability per 250 mL sample a constant per mL probability, then our ability to detect ranavirus in a sample varied from a low of 1-(1–0.90)^75mL/250mL^ = 0.499 to an average of 1-(1–0.90)^6000mL/250mL^ = 0.99 or higher per water body. Hall et al.’s estimate comes from ponds with active die-offs, so these estimates of detection probability are likely high. For *Bd*, Schmidt et al. (2013) [44] found that each 600 mL water sample from high-elevation ponds in Arizona had a detection probability of 0.45 when present in amphibians. Although they used a different sampling design (20 mL from 30 locations in a pond) and filters (0.22 μm polyvinylidene difluoride filters), if we use their estimate as a first approximation, then our probability of detecting *Bd* in body of water would have varied with the amount of water filtered per sample from a low of 1-(1–0.45)^75mL/600mL^ = 0.072 to a high of 1-(1–0.45)^6000mL/600mL^ = 0.997, although on average it would have been closer to 1-(1–0.45)^500mL/600mL^ = 0.392 per water body. It is possible that low pathogen density and/or high heterogeneity in the distribution of *Bd* may be responsible for the low detection probabilities previously reported, but the absence of *Bd* in all our 68 independent water samples does bolster the conclusion from the swab data: *Bd* was rare or absent in much of Madagascar during this survey. Furthermore, since we specifically targeted locations expected to favor pathogen presence, the lack of detection in our pathogen-negative samples provides greater certainty of pathogen absence than would similar results produced by a random sampling effort.

Our field results suggest that *Bd* and ranavirus were absent from most regions sampled in Madagascar at the time surveillance was performed, but whether these pathogens follow seasonal patterns in Madagascar warrants further investigation. Prevalence, infection intensity, and timing of disease-associated mortality events can fluctuate seasonally; some *Bd* surveys have demonstrated greater infection prevalence in cooler versus warmer months [27,45–48], whereas ranavirus appears to become more active in summer [8,49]. Seasonal patterns in adult infection with *Bd* (and perhaps ranavirus) appear to be largely temperature-driven, and the environmental conditions we recorded during this investigation fell near those optimal for growth and reproduction in culture, for both pathogens (average water and air temperatures measured 23.3 C and 21.5 C, respectively). Exposure to 32 C for four hours is lethal to *Bd* [27] and replication of the type ranavirus, FV3 ceases at 33 C [28]. Although 43/45 aquatic bodies measured less than 32 C when water was sampled during daylight hours, it is conceivable that hostile conditions may have occurred prior to our visit and influenced pathogen abundance and detectability during our survey, as suggested by Murray et al. (2013) [48]. Still, Chestnut et al. (2014) [50] investigated temporal patterns of *Bd* presence in a wetland in Oregon, USA and found that *Bd* remained detectable by water filtration year-round, including the warmer periods when fewer adult animals sometimes test positive for infection. Similarly, both Whitfield et al. (2012) [47] and Longo et al. (2010) [51] detected *Bd*-positive amphibians year-round via skin swabbing in a warm lowland site in Costa Rica and a cool upland forest in Puerto Rico, respectively, even during the warmest months when infection loads were at their lowest. Therefore, despite potentially low pathogen abundance during our survey in Madagascar, these data suggest that our *Bd*-negative water filter results together with *Bd*-negative amphibian swab results is more likely demonstrative of pathogen absence from a location rather than a seasonal false-negative characterization. This is especially relevant to our sampling efforts at cooler high elevation sites, such as Ankaratra (2,015–2,384 m elevation; Table 1), where conditions are unlikely to exceed the thermal maximum for pathogen survival, even through summer. Regardless, a series of amphibian and environmental surveys should be repeated at our sites to determine whether the strains of *Bd* and/or ranavirus present in Madagascar exhibit seasonal variation in prevalence and aquatic abundance and to what magnitude this may manifest in field results.

We previously detected *Bd* in 3 of 565 (prevalence = 0.5%; 95% CI: 0.2–1.6%) wild-collected amphibians exported from Madagascar in February 2012, although due to the possibility of exotic trade-associated contamination whilst in temporary captivity prior to export, it was difficult to confirm whether these animals were collected from the wild with *Bd* at that time [17]. Therefore, during this field study we performed an inspection at the specific wildlife trade facility in Toamasina from which the amphibians positive for *Bd* (and ranavirus) had been exported, and conducted interviews with the staff. We were unable to identify any potential sources of non-Malagasy *Bd* introduction from within this compound. Only amphibians, reptiles, and birds collected from the wild in Madagascar were temporarily housed at this facility and no exotic animals were received. Furthermore, this trade enterprise did not import or transship fresh produce or aquacultural material that might have inadvertently introduced amphibians or their pathogens. Therefore, we suspect the infected, exported amphibians reported in Kolby (2014) [17] were indeed collected from the wild in Madagascar with *Bd*.

Further, the absence of *Bd* detection in the present study does not, in fact, contradict our previous finding, assuming these exported animals had become infected in the wild. Indeed, the 95% confidence interval for *Bd* prevalence in the current study (0–0.8%) overlaps with that from Kolby (2014) [17] of 0.2–1.6%, suggesting there may have been undetected low infection prevalence in the wild during the current investigation of similar magnitude as that previously found in the exported frogs. Furthermore, combining species in high density at the trade facility prior to export was likely to have increased the opportunity for disease transmission, suggesting that true *Bd* prevalence in the source population(s) was closer to 0.2% before collection, a challenging prevalence to identify in the field especially when the location of these animals' collection remains unknown.

The detection of ranavirus in wild amphibians in Ranomafana National Park and Ankaratra, and in wildlife trade facilities in Antananarivo and Toamasina, demonstrates a potential threat to the amphibian biodiversity in these and surrounding regions. Ranavirus infection in amphibians can result in unpredictable mass mortality events, dramatic population decline, and/or local extirpation [3,4,52], and it is unknown whether the ranavirus we detected is highly virulent to native species. The surveys we performed at each site were brief and designed to identify pathogen presence, but not mortality. It is therefore possible that our single visits per location failed to capture disease-associated mortality events, especially if a highly virulent ranavirus was present that induced rapid mortality at other times. Of particular concern is the presence of ranavirus at Ankaratra, a remote area inhabited by two locally endemic critically endangered amphibian species: *Boophis williamsi* and *Mantidactylus pauliani*, the former of which is regarded to be one of the most threatened amphibians in Madagascar according to the IUCN Red List of Threatened Species (2014) [53]. Likewise true with respect to *Bd*, sufficient information to discern whether the ranavirus we detected was recently introduced or endemic to Madagascar is not available at this time. Accordingly, standardized long-term population surveys to monitor the potential impact of ranaviral infection in these two highly vulnerable species should be established with urgency.

Additional biodiversity hotspots oriented near ranavirus-positive locations, such as Andasibe-Mantadia National Park, Zahamena National Park, and Betampona Strict Nature Reserve, are threatened by pathogen exposure and warrant additional monitoring. Both Ranomafana and Andasibe are frequently visited by tourists and researchers and the movement of potentially contaminated footwear and equipment provides a likely vector for the spread of disease among and between biodiversity hotspots. Ranavirus can remain viable from days to weeks when protected from high temperatures, desiccation, and microbial action [54,55] providing considerable time for spread via fomites, and the same applies to *Bd* [56]. Even if these pathogens may be endemic to Madagascar, human-assisted introduction to naive isolated amphibian populations or the spread of different strains to new regions may lead to more severe outcomes and increased risk of declines. Disinfection of materials exposed to amphibians or aquatic habitats is necessary to prevent increased rates of disease spread [38], and a variety of commercially available disinfectants will inactivate *Bd* and ranavirus, including bleach at 3.0% concentration or higher [57,58]. Accordingly, public education and vigilance are critically important to reduce the frequency of accidental pathogen dispersal beyond current boundaries. Furthermore, it would be prudent for Malagasy authorities to require biosecurity protocols be performed at wildlife trade facilities to reduce the risk of pathogen spillover. Since animals from disparate regions become centralized at these locations prior to exportation, the untreated disposal of pathogen-positive water, soil, or dead animals from temporary housing enclosures may expose amphibians living in proximity to centers of wildlife trade and accelerate the spread of *Bd* and ranavirus within Madagascar. Further, we cumulatively detected both ranavirus and *Bd* at the same trade facility in Toamasina, suggesting that co-infection may particularly threaten free-ranging amphibian populations near trade centers that do not employ biosecurity measures.

The current National Monitoring Plan (NMP) for the detection of *Bd* in Madagascar involves biannual swabbing surveys performed at eight fixed locations throughout the country [14], but early detection is more likely if surveillance is broadened to include additional locations, species, and sampling methods. Recognizing resource limitations and how potential seasonal influences on *Bd* might promote detectability in adult amphibians during cooler temperatures, an efficient approach would involve doubling the number of national sampling points but reducing sampling frequency to once per year, redirecting all survey efforts to the winter season. Further, the incorporation of water filtration methods would provide cost-efficient preliminary screening of amphibian habitats to detect pathogen presence before performing intensive amphibian swabbing surveys to identify infection prevalence. Regions affected by alien invasive species that might provide a vector for amphibian pathogen introduction should also be included in this sampling regime, specifically Toamasina, where an incursion of Asian common toads (*Duttaphrynus melanostictus*) was recently identified [59]. Lastly, our detection of ranavirus at both wildlife export facilities sampled demonstrates a need for the development of cooperative efforts between Malagasy authorities and these facilities. Disclosure of amphibian collection localities and permission to test the large numbers of amphibians amassed prior to export could help accelerate field detection by focusing NMP field efforts towards regions where wild populations may already be affected by *Bd* and/or ranavirus.

Ranavirus can also infect and cause disease and mortality in reptiles, especially chelonians, and transmission of novel strains from amphibians may threaten Madagascar's endangered reptile species [16, 60,61]. Nearly 40% of the country's reptile diversity faces a high risk of extinction primarily due to habitat loss and exploitation for food or pets [62]. Fortunately, we did not detect the presence of ranavirus in water sampled from enclosures of critically endangered Ploughshare tortoises (*Astrochelys yniphora*) and Madagascar big-headed turtles (*Erymnochelys madagascariensis*) at the Durrell Chelonian Captive Breeding Centre (0 of 3 samples; Table 4), but increased vigilance would benefit such reptile conservation efforts. Despite strict biosecurity measures taken by staff to prevent introduction of pathogens via their own movements, animals bred in captivity and maintained in outdoor enclosures may become exposed to ranavirus through incidental contact with local herpetofauna if able to pass through or over fences, and as demonstrated by Brenes et al. (2014) [16], transmission can occur through water shared between amphibians and reptiles. Even endemic strains of ranavirus can cause periodic mortality and threaten species that exist only in isolated limited numbers. Although ranaviral infection in reptiles is most often identified in chelonians, Malagasy lizards of conservation concern, such as *Uroplatus* spp. geckos, may also be susceptible and warrant attention [63]. Our detection of ranavirus in free-ranging amphibians sampled during this investigation illustrates the need for preemptive surveillance among endangered and range-restricted reptile species to evaluate this potential threat.

On the morning of 26 February 2014, we recorded an enigmatic amphibian mortality event in Analamay, where 20 dead frogs (*Heterixalus* spp.) were found in a shallow pool of rainwater on a dirt road that passed through an area of forest. We were alerted to the scene and collected water filter samples for pathogen presence that same day. Unfortunately, all frog carcasses displayed advanced stages of decomposition and were unsuitable for pathological examination. However, on 3 March 2014, four additional dead frogs (*Aglyptodactylus* sp. (n = 1) and *Heterixalus* spp. (n = 3)) were found in better condition in or near the same pool, and preserved for histological and molecular analysis. All samples collected from this location tested negative for the presence of *Bd* and ranavirus both by qPCR and histology and no lesions suggestive of either chytridiomycosis or ranaviral disease were observed. The potential presence of other pathogens, environmental contaminants, or habitat degradation, might have contributed towards this mortality event, but the precise cause(s) remains unidentified. Due to the differences in sampling conditions and quality, these data are not combined with those summarized in Tables 1–3. Populations of the critically endangered Golden Mantella (*Mantella aurantiaca*) inhabit this forest area, raising concern for additional mortality events in the region. This particular event marks the first amphibian mass mortality reported to the Madagascar Chytrid Emergency Cell for rapid investigation and any future events should be similarly reported and evaluated.

At the time this rapid response investigation was performed, presence of Bd in wild amphibian populations in Madagascar had not yet been confirmed, and was still only suggested by the 2012 detection in exported amphibians [17]. Immediately prior to the publication of this report, Bletz et al. (2015) [64] described current widespread Bd presence in Madagascar, and included records of detection in field samples that dated back to 2010. These data highly contradict those reported here collected in early 2014. Detection of Bd at locations in Madagascar as reported by Bletz et al. [64] was inconsistent. This, together with the fact that multiple sampling and diagnostic methods with variable accuracy have been used, means that the establishment of the pathogen in Madagascar cannot yet be described with certainty from existing data. Accordingly, before prematurely responding with emergency conservation rescue initiatives that could misdirect limited conservation resources, the accuracy and context of all existing data now warrants cautious review to resolve the discordance between field survey results. Additionally, increased coordination and standardization between the Bd field surveys of Madagascar’s National Monitoring Plan are imperative in order to mitigate further challenges in responding to this potential biodiversity crisis.

Although we did not detect *Bd*-positive amphibians in Madagascar, the risk-based approach of our field surveillance activity suggests that the greatest threat posed by chytridiomycosis likely remains confined to limited regions and/or seasonal periods. Further work to identify the strain(s) of *Bd* present is needed to evaluate the risk of decline posed to native species and whether the commonly used diagnostic PCR method fails to detect a potentially highly divergent Malagasy *Bd* strain. Similarly, the distribution and dynamics of ranavirus in Madagascar, and whether it is endemic or a recent introduction, requires additional field surveillance to resolve. Therefore, it is important that standardized population monitoring of key amphibian and reptile populations be established with urgency to enable early detection of potential impacts of disease emergence in this global biodiversity hotspot before obvious declines are observed [7,64]. Risk assessments should include prediction of pathogen impacts in various habitats and prioritization of species based on their ecology and results from infection susceptibility trials [20,65,66]. Fortunately, the establishment of amphibian captive breeding initiatives by Association Mitsinjo and Madagascar Fauna and Flora Group preempted formal identification of amphibian pathogens in the country and are developing local capacity to respond with rescue activities if needed. We remain hopeful that disease-driven amphibian extinction can be prevented in Madagascar through continued monitoring of *Bd* and ranavirus distribution and spread, accurate predictions of disease impacts, and coordinated field and ex-situ management activities.
