# Correction: Rapid Response to Evaluate the Presence of Amphibian Chytrid Fungus (*Batrachochytrium dendrobatidis*) and Ranavirus in Wild Amphibian Populations in Madagascar

**DOI:** 10.1371/journal.pone.0134524

**Published:** 2015-07-29

**Authors:** Jonathan E. Kolby, Kristine M. Smith, Sara D. Ramirez, Falitiana Rabemananjara, Allan P. Pessier, Jesse L. Brunner, Caren S. Goldberg, Lee Berger, Lee F. Skerratt

There are a number of errors in the caption for Fig 1, “Locations sampled for the presence of *Bd* and ranavirus in Madagascar.” Please see the complete, correct Fig 1 caption here.

**Fig 1:**
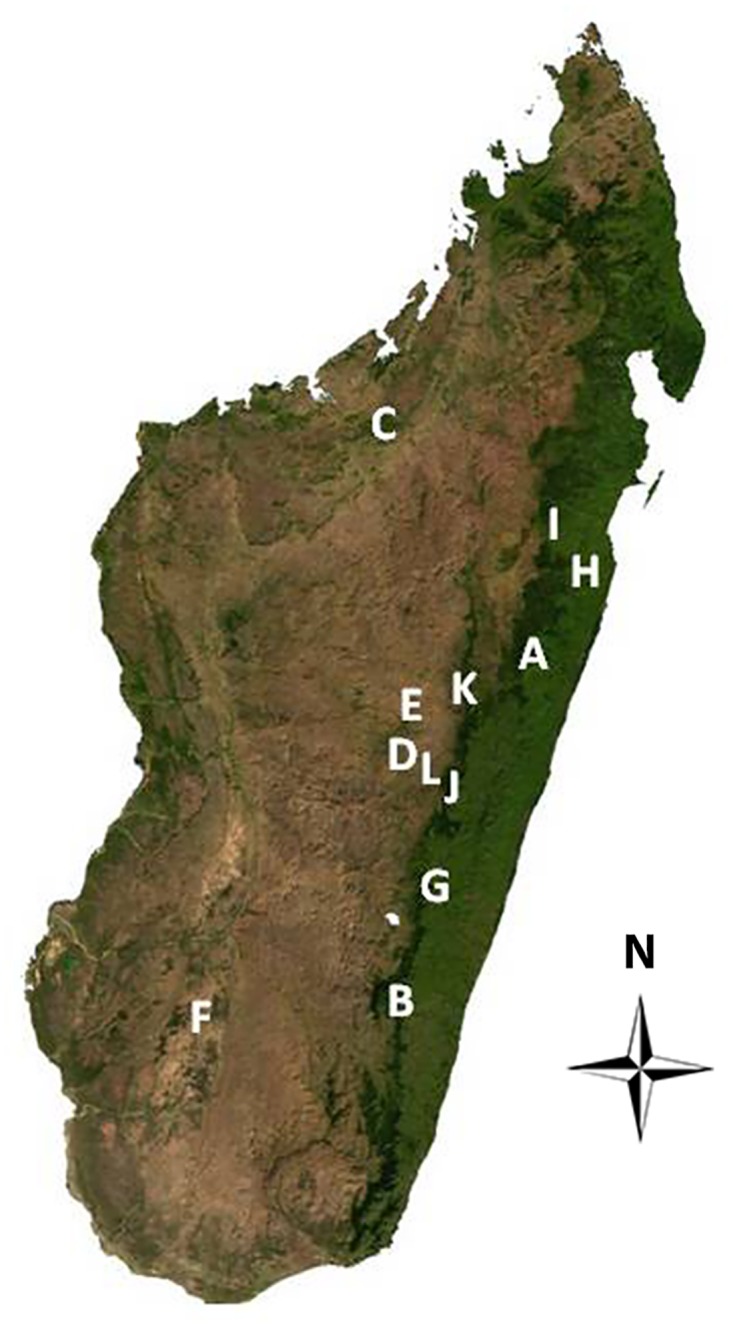
Locations sampled for the presence of *Bd* and ranavirus in Madagascar. Ambatolampy (J), Analamay (K), Andasibe (A), Andringitra National Park (B), Ankarafantsika (C), Ankaratra (D), Antananarivo (E), Faravohitra (L), Isalo (F), Ranomafana National Park (G), Toamasina (H), Zahamena National Park (I).
